# Resolving degeneracy in diffusion MRI biophysical model parameter estimation using double diffusion encoding

**DOI:** 10.1002/mrm.27714

**Published:** 2019-03-13

**Authors:** Santiago Coelho, Jose M. Pozo, Sune N. Jespersen, Derek K. Jones, Alejandro F. Frangi

**Affiliations:** ^1^ Centre for Computational Imaging & Simulation Technologies in Biomedicine (CISTIB) and Leeds Institute for Cardiac and Metabolic Medicine (LICAMM), School of Computing & School of Medicine University of Leeds Leeds United Kingdom; ^2^ CISTIB, Electronic and Electrical Engineering Department The University of Sheffield Sheffield United Kingdom; ^3^ Center of Functionally Integrative Neuroscience (CFIN) and MINDLab, Department of Clinical Medicine Aarhus University Aarhus Denmark; ^4^ Department of Physics and Astronomy Aarhus University Aarhus Denmark; ^5^ Cardiff University Brain Research Imaging Centre (CUBRIC) Cardiff University Cardiff United Kingdom; ^6^ School of Psychology Australian Catholic University Melbourne Australia

**Keywords:** biophysical tissue models, diffusion MRI, double diffusion encoding, microstructure imaging, parameter estimation, single diffusion encoding, white matter

## Abstract

**Purpose:**

Biophysical tissue models are increasingly used in the interpretation of diffusion MRI (dMRI) data, with the potential to provide specific biomarkers of brain microstructural changes. However, it has been shown recently that, in the general Standard Model, parameter estimation from dMRI data is ill‐conditioned even when very high b‐values are applied. We analyze this issue for the Neurite Orientation Dispersion and Density Imaging with Diffusivity Assessment (NODDIDA) model and demonstrate that its extension from single diffusion encoding (SDE) to double diffusion encoding (DDE) resolves the ill‐posedness for intermediate diffusion weightings, producing an increase in accuracy and precision of the parameter estimation.

**Methods:**

We analyze theoretically the cumulant expansion up to fourth order in b of SDE and DDE signals. Additionally, we perform *in silico* experiments to compare SDE and DDE capabilities under similar noise conditions.

**Results:**

We prove analytically that DDE provides invariant information non‐accessible from SDE, which makes the NODDIDA parameter estimation injective. The *in silico* experiments show that DDE reduces the bias and mean square error of the estimation along the whole feasible region of 5D model parameter space.

**Conclusions:**

DDE adds additional information for estimating the model parameters, unexplored by SDE. We show, as an example, that this is sufficient to solve the previously reported degeneracies in the NODDIDA model parameter estimation.

## 1 INTRODUCTION

Diffusion MRI (dMRI) has been established as an invaluable tool for characterizing brain microstructure *in vivo* and non‐invasively. Diffusion weighted images (DWIs) are sensitive to the random displacement of water molecules within a voxel,^1^ probing tissue on scales considerably lower than image resolution.^2^ Diffusion MRI provides the aggregate signal from the distribution of components within a voxel. By measuring across multiple diffusion orientations and weightings, information about the underlying tissue architecture can be unravelled. The ability to detect small alterations in brain tissue is a key factor when developing biomarkers for early stages of neurodegenerative diseases.^3^ Various approaches to derive information from Diffusion Weighted Images (DWI) have been proposed in the literature.^4^, ^5^, ^6^, ^7^, ^8^ Most direct approaches, such as Diffusion Tensor Imaging (DTI),^4^ are just aimed at describing the main MRI signal characteristics (signal representations,^9^). However, the quest for specific information on tissue microstructural integrity inspired the development of biophysical tissue models.^10^, ^11^, ^12^, ^13^ By assuming certain characteristics for the tissue, such as the type of constituents, their geometry and physical properties, these models may allow the extraction of more specific microstructural information than signal representations, as long as these assumptions are at least approximately satisfied by the tissue. Nevertheless, the validity of these results relies on how accurate the model is for the tissue under study. The widely used Neurite Orientation Dispersion and Density Imaging (NODDI)^14^ model fixes the diffusivity values of the compartments present in the voxel to specific values. NODDI’s assumptions have been shown to be incompatible with data from spherical tensor encoding (STE) in Lampinen et al^15^ and it has been argued to introduce bias in the estimation of the remaining model parameters.^16^ To overcome this limitation, Jelescu et al^17^ extended the model by adding the diffusivities to the estimation routine, and removing the CSF compartment. They dubbed it NODDIDA (NODDI with Diffusivity Assessment). While this approach eliminated some flawed assumptions made by NODDI, this led to multiple possible solutions that describe the signal equally well. This reflects that the estimation problem is ill‐posed or, at least, ill‐conditioned, and is usually stated as the existence of degenerated model parameter sets. Recent work by Novikov et al showed that this degeneracy is intrinsic to the so‐called Standard Model (SM),^18^ of which NODDIDA is a special case. They show that choosing the correct solution is challenging even with the use of high b‐value data, although Jespersen et al^19^ obtained stable estimations in ex‐vivo brain tissue using extremely high b‐values (15 ms/$\mu {\text{m}}^{2}$). Reisert et al^20^ proposed a supervised machine learning approach trained with the expected value of the Bayesian posterior, which, by definition, disregards the possible multimodality of the distribution. Furthermore, it was trained on simulated data with the prior assumption of similar traces for the intra‐ and extra‐axonal diffusivities.

Most of the dMRI techniques have been developed for an acquisition performed within a Single Diffusion Encoding (SDE) framework. Since Stejskal and Tanner developed the Pulsed Gradient Spin Echo (PGSE) sequence,^21^ there have been many works aimed at maximizing the information that can be obtained from a dMRI experiment by exploring different acquisition protocols.^22^, ^23^ One of the many modifications proposed to the magnetic gradient waveforms involves the addition of multiple gradient pairs. Particularly, a scheme that has lately gained popularity is termed double diffusion encoding (DDE),^24^ first proposed by Cory et al.^25^ The term DDE refers to any sequence consisting of two consecutive diffusion encodings. It has been shown that DDE, as well as other multiple encoding schemes, has the potential to provide new information that is not immediately accessible with SDE.^26^ Many groups focused on developing methods for extracting microstructural information based on this scheme.^27^, ^28^, ^29^, ^30^ Jespersen et al^31^ showed that in the low‐diffusion‐weighting limit, the information extracted from single and multiple diffusion encodings is the same. A recently developed dMRI framework based on q‐space trajectory encoding (i.e. multidimensional diffusion encoding) was proposed to probe tissue in ways not accessible by SDE.^32^ For tissues comprised of multiple Gaussian compartments (MGCs) any q‐space trajectory is equivalent to a second order b‐tensor, which generalizes the concept of b‐value. In such systems SDE and DDE are fully specified by b‐tensors, with one and two non‐zero eigenvalues, respectively, and are also called linear tensor encoding (LTE) and planar tensor encoding (PTE), in case of DDE with perpendicular directions. Lampinen et al^15^ have analyzed the advantages of a multidimensional encoding over SDE NODDI. They proved that extending the acquisition increases the accuracy in quantifying microscopic anisotropy. However, it has not been fully explored, from the point of view of fitting a biophysical model to noisy measurements, if single or multiple encodings can provide us with more precise model parameter estimates (cf.^29^, ^30^). Recently, the advantages of combining linear with planar or spherical tensor encoding to address the degeneracy and increase the precision of parameter estimation have been investigated^33^, ^34^, ^35^ in both *in silico* and/or *in vivo* experiments. Their results show that the estimation precision is increased by the addition of these orthogonal measurements. However, a theoretical background of why this happens is still missing.

This paper extends NODDIDA to a DDE scheme and assesses the accuracy of estimators based on SDE and DDE measurements. This extension adds more degrees of freedom to the data acquisition (i.e. two diffusion encoding periods must be chosen). We hypothesized that DDE acquisition protocols containing both parallel and perpendicular direction pairs might outperform SDE protocols in informing biophysical models. We investigated analytically the different information provided by DDE and SDE in terms of their fourth order cumulant expansions. We examine the ill‐posed nature of the parameter estimation from SDE and present a theoretical explanation of why DDE resolves the degeneracy (except for the completely isotropic case *κ* = 0) without requiring extremely high diffusion weightings (e.g. $b\;>\;4\;\text{ms}/\mu {\text{m}}^{2}$). Additionally, we generated *in silico* dMRI measurements for acquisitions with different DDE configurations from a wide range of model parameter values covering the biologically feasible region of the 5D parameter space. Under similar experimental conditions, higher accuracy and precision is obtained for DDE combining parallel and perpendicular direction pairs, outperforming SDE in most scenarios.

## 2 THEORY

### 2.1 Biophysical model assumptions

A general assumption among multi‐compartment models representing tissue microstructure is that water exchange between compartments is negligible for typical experimental time scales. The total signal is the weighted contribution from each compartment. The two‐compartment model dubbed *Standard Model* is the most general version of the typical models used for diffusion in neural tissue (see Ref.^36^). The stick compartment (sometimes referred as intra‐neurite) represents axons, which are expected to be the main contributors to the restricted diffusion signal, and, possibly, dendrites and glial processes.^19^ The inclusion of dendrites and glial processes is open to discussion^36^ and implies the assumption that in certain regimes (depending on e.g. diffusion time) they have similar diffusivity and $T_{2}$ relaxation properties and directional distribution, a question which still has not been fully addressed (see partial discussion in Lampinen et al^37^). Sticks are zero‐radius cylinders and model fibers in which diffusion is assumed to occur only along the fiber’s main direction as it was first proposed for water in neurites in Jespersen et al.^13^ Later, Nilsson et al^38^ showed theoretically that typical axonal diameters cannot be resolved with SDE and gradient amplitudes available on clinical scanners and thus, are indistinguishable from sticks. This was also confirmed experimentally in Veraart et al.^39^ The second compartment represents the extra‐neurite space where diffusion is hindered and is modeled as Gaussian anisotropic^19^ (zeppelin compartment). A *fiber segment* is defined as the local bundle of aligned sticks with the extra‐neurite space surrounding them. Voxels are composed of a large number of fiber segments. The SM consists of the fiber segment signal model (i.e. kernel) with the diffusivities and water fraction as free parameters, together with a general fiber orientation distribution function (ODF), which could be represented by its spherical harmonics decomposition. One limitation of this model is that each fascicle within a voxel is assumed to have identical diffusion properties, leading to identical microstructural parameters.

Some other works consider a third compartment that represents the contribution from stationary water.^11^, ^40^ However, recent works^41^ have concluded that the signal arising from this compartment can be neglected in most structures for the diffusion times used in the clinic and should only be considered in the cerebellum.^42^ Additionally, in its original version, NODDI included an isotropic diffusion compartment to account for the presence of cerebrospinal fluid (CSF). This compartment was removed from NODDIDA for the sake of simplicity.^17^

**Figure 1:**
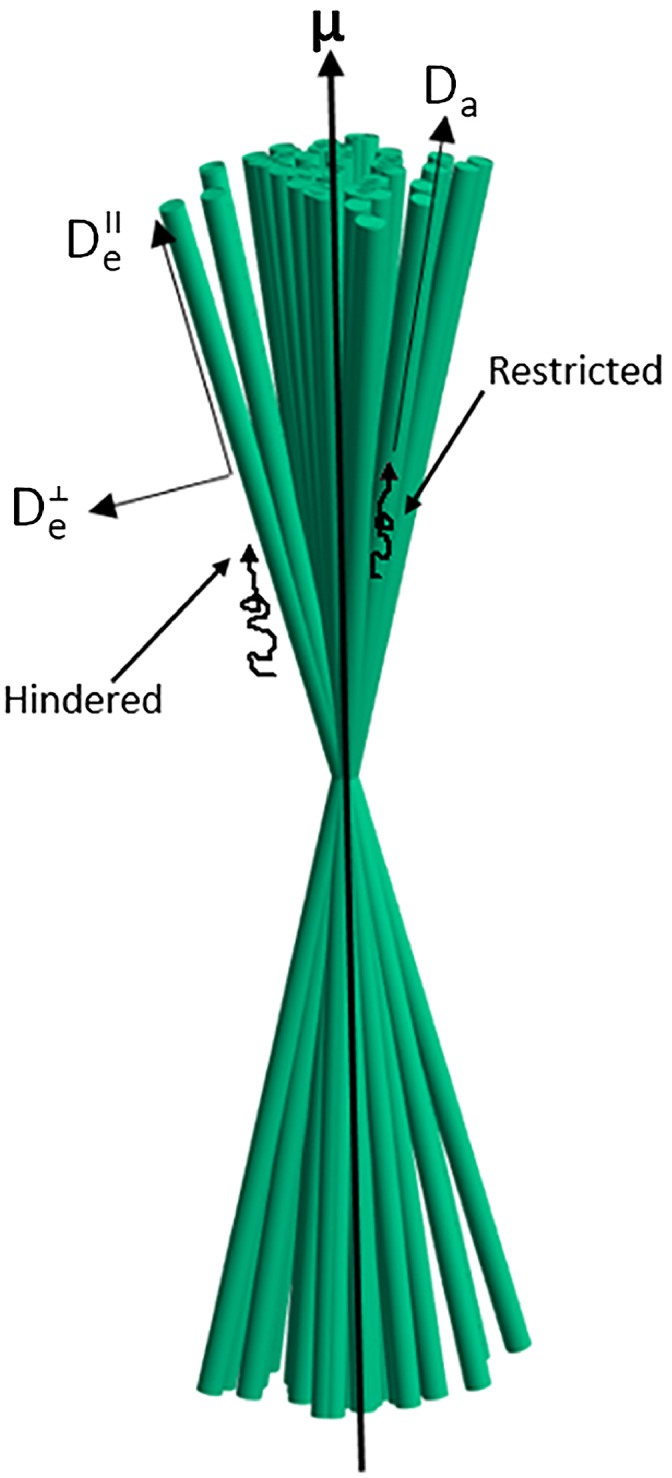
Diagram of the two compartments present in the NODDIDA tissue model with their corresponding diffusivities

Considering a general fiber ODF involves a large set of parameters, which can hinder their unambiguous estimation from the dMRI signal. The NODDIDA model,^17^ is essentially the SM with the constraint that the fiber ODF must be a Watson spherical distribution $P\left(\hat{u}\right)\;=\;f\left(\hat{u}|\hat{\mu },\;\kappa \right)$, with concentration parameter *κ* and main direction $\hat{\mu }$ (see Figure 1). This cylindrically symmetric ODF is usually considered a sufficiently good and parsimonious model,^43^ especially for white matter regions without crossing fibers. Although being a simplified version of SM, NODDIDA still presents some degeneracy problems. Thus, in this work, we focus our analysis on the NODIDDA model.

### 2.2 NODDIDA model with SDE

For a general SM, the signal from a SDE experiment, where the diffusion weighting *b* (i.e. b‐value) is applied in the diffusion encoding direction $\hat{n}\;=\;{\left[n_{x},\;n_{y},\;n_{z}\right]}^{t}$, is given by the convolution over the unit sphere^18^

(1) $$S_{\mathrm{SDE}}\left(b,\;\hat{n}\right)=S_{0}\int_{S^{2}}P\left(\hat{u}\right)K\left(b,\;\hat{n}\cdot \hat{u}\right)dS_{\hat{u}},$$

where

(2) $$K\left(b,\;\hat{n}\cdot \hat{u}\right)\;=\;f\exp\left[-bD_{a}{\left(\hat{n}\cdot \hat{u}\right)}^{2}\right]+\left(1-f\right)\exp\left[-bD_{e}^{⊥}-b\Delta_{e}{\left(\hat{n}\cdot \hat{u}\right)}^{2}\right],$$

is the response signal (kernel) from a fiber segment oriented along direction $\hat{u}$. Here, *f* is the (mainly) $T_{2}$‐weighted stick volume fraction, $D_{a}$ the intra‐neurite axial diffusivity, and $\Delta_{e}\;=\;D_{e}^{\left|\right|}-D_{\text{e}}^{⊥}$, with $D_{e}^{\|}$, $D_{e}^{⊥}$ the extra‐neurite diffusivities parallel and perpendicular to the fiber‐segment axis.^36^ These scalar kernel parameters (*f*, $D_{a}$, $D_{e}^{\|}$, and $D_{e}^{⊥}$) provide important tissue microstructural information, and have shown potential clinical relevance as they are sensitive to specific disease processes such as demyelination, axonal loss or inflammation.^44^, ^45^, ^46^

It has been recently shown that the parameter estimation is challenging under normal experimental conditions.^17^ There are two issues here. The first one is that fitting these models to noisy measurements is generally a non‐convex optimization problem, potentially having several local minima of the objective function, requiring appropriate optimization algorithms. However, the existence of multiple local minima opens the door to a second, more serious, issue: the objective function can present multiple minima with equal or very similar values. In the presence of noise these minima are perturbed, making unstable which one becomes the global minimum. Jelescu et al^17^ evidenced this ill‐posedness issue for clinically feasible dMRI acquisitions in two particular cases. They showed that the estimated parameters from a collection of independently simulated dMRI measurements follow a bi‐modal distribution, despite being simulated from a single ground truth, and the presence of practically indistinguishable spurious minima in the objective function.

### 2.3 Parameter estimation from SDE: An ill‐posed problem

A recent work by Novikov et al^18^ analyzed in detail this inverse problem for the unconstrained SM by reparametrizing it into its rotational invariants. They concluded that without any constraints on the ODF shape, it was not possible to estimate the kernel parameters with an acquisition sensitive up to order $O(b^{2})$. However, in this work we are interested in studying NODDIDA, where the ODF is given by a Watson distribution.

For intermediate diffusion weightings (i.e. $b\;<\;2.5\;\text{ms}/\mu {\text{m}}^{2}$) the dMRI signal is accurately represented by its $4^{\mathrm{th}}$‐order cumulant expansion^47^ (sensitive up to $O(b^{2})$ contributions). For SDE this expansion can be written as^8^

(3) $$\begin{array}{cc} \log(S\left(b,\;\hat{n}\right)/S_{0})\; & \approx -bn_{i}n_{j}D_{\mathrm{ij}}+\frac{1}{6}b^{2}{\overset{¯}{D}}^{2}n_{i}n_{j}n_{k}n_{\ell }W_{ijk\ell }\\ & =-bD\left(\hat{n}\right)+\frac{1}{6}b^{2}{\overset{¯}{D}}^{2}W\left(\hat{n}\right), \end{array}$$

where $S_{0}\;=\;S\left(b\;=\;0\right)$ is the unweighted signal, **D** and **W** are the diffusion and kurtosis tensors, respectively, with $\overset{¯}{D}\;=\;tr(D)$, as defined in Hansen et al^48^ and Einstein’s summation convention is implied. Let us consider a voxel with fibers oriented according to a Watson ODF. Following an analogous procedure as in Novikov et al^18^ we can expand the signal $S(b,\;\hat{n})$ in Equation 1 up to order $O(b^{2})$ according to Equation 3. This gives a mapping between the biophysical parameter (BP) space and the diffusion kurtosis (DK) space, removing the dependence with the acquisition settings and simplifying the analysis of whether different sets of model parameters produce the same signal profile.

Due to the axial symmetry of the Watson distribution, the corresponding diffusion and kurtosis tensors can be expressed in terms of the projection, $\xi \;=\;\hat{n}\cdot \hat{\mu }$, of the gradient direction to the main direction $\hat{\mu }$ Jespersen et al^49^, ^1^:

(4) $$\begin{array}{cc} D(\xi )\;=\; & \left(fD_{a}+\left(1-f\right)\Delta_{e}\right)h_{2}\left(\xi ,\kappa \right)+\left(1-f\right)D_{e}^{⊥},\\ W\left(\xi \right){\overset{¯}{D}}^{2}\;=\; & 3\left[\left(fD_{a}^{2}+\left(1-f\right)\Delta_{e}^{2}\right)h_{4}\left(\xi ,\kappa \right)+2\left(1-f\right)\Delta_{e}D_{e}^{⊥}h_{2}\left(\xi ,\kappa \right)+\left(1-f\right)D_{e}^{⊥2}-D{\left(\xi \right)}^{2}\right], \end{array}$$

where $h_{2}\left(\xi ,\;\kappa \right)\;=\;\frac{1}{3}+\frac{2}{3}p_{2}P_{2}\left(\xi \right)$ and $h_{4}\left(\xi ,\;\kappa \right)\;=\;\frac{1}{5}+\frac{4}{7}p_{2}P_{2}\left(\xi \right)+\frac{8}{35}p_{4}P_{4}\left(\xi \right)$ are defined as in Jespersen et al^49^ $P_{2}\left(\xi \right)$ and $P_{4}\left(\xi \right)$ are the second and fourth order Legendre polynomials, and $p_{2}$, $p_{4}$ the non‐zero second and fourth order coefficients of the spherical harmonics expansion of the Watson distribution:

(5) $$\begin{array}{cc} p_{2} & =\frac{1}{4}\left[\frac{3}{\sqrt{\kappa }F\left(\sqrt{\kappa }\right)}-2-\frac{3}{\kappa }\right],\\ p_{4} & =\frac{1}{32\kappa^{2}}\left[105+12\kappa \left(5+\kappa \right)+\frac{5\sqrt{\kappa }\left(2\kappa -21\right)}{F(\sqrt{\kappa })}\right], \end{array}$$

where *F* denotes the Dawson function.^50^ Using these equations, we can derive the relations between the BP and DK parameters that fully describe this axially symmetric environment, as done in Hansen et al^51^ for fully aligned fibers, but here for an arbitrary value of *κ*:

(6) $$\begin{array}{cc} D_{\|} & \;=\;\left(fD_{a}+\left(1-f\right)\Delta_{e}\right)h_{2}\left(1,\kappa \right)+\left(1-f\right)D_{e}^{⊥},\\ D_{⊥} & \;=\;\left(fD_{a}+\left(1-f\right)\Delta_{e}\right)h_{2}\left(0,\kappa \right)+\left(1-f\right)D_{e}^{⊥},\\ \frac{1}{3}W_{\|}{\overset{¯}{D}}^{2}+D_{\|}^{2} & \;=\;\left(fD_{a}^{2}+\left(1-f\right)\Delta_{e}^{2}\right)h_{4}\left(1,\kappa \right)+2\left(1-f\right)\Delta_{e}D_{e}^{⊥}h_{2}\left(1,\kappa \right)+\left(1-f\right)D_{e}^{⊥2},\\ \frac{1}{3}W_{⊥}{\overset{¯}{D}}^{2}+D_{⊥}^{2} & \;=\;\left(fD_{a}^{2}+\left(1-f\right)\Delta_{e}^{2}\right)h_{4}\left(0,\kappa \right)+2\left(1-f\right)\Delta_{e}D_{e}^{⊥}h_{2}\left(0,\kappa \right)+\left(1-f\right)D_{e}^{⊥2},\\ \frac{5\overset{¯}{W}{\overset{¯}{D}}^{2}}{8}-\frac{W_{⊥}{\overset{¯}{D}}^{2}}{4}-\frac{W_{\|}{\overset{¯}{D}}^{2}}{24}+\frac{{\left(D_{⊥}+D_{\|}\right)}^{2}}{4} & \;=\;\left(fD_{a}^{2}+\left(1-f\right)\Delta_{e}^{2}\right)h_{4}\left(\frac{1}{\sqrt{2}},\kappa \right)+2\left(1-f\right)\Delta_{e}D_{e}^{⊥}h_{2}\left(\frac{1}{\sqrt{2}},\kappa \right)+\left(1-f\right)D_{e}^{⊥2}, \end{array}$$

where $\overset{¯}{D}\;=\;(2D_{⊥}\;+\;D_{\|})/3$. Taking the limit for *κ*→∞ we recover the system of equations for parallel fibers presented in Hansen et al^51^ (Equation 12).

In Hansen et al^48^ the equivalent to the system in Equation 6 is solved reaching two alternative equations for *κ*, $F_{\pm }\left(\kappa \right)\;=\;0$, each giving possible solutions. This suggested that, in general, there should be two solutions, one for each branch. However, this is not always the case, as illustrated in Table 1. We derive here an alternative expression of the solution in one equation only. First, Equation 6 can be reparametrized as:

(7) $$\begin{array}{cc} \alpha \; & =\;fD_{a}+\left(1-f\right)\Delta_{e},\beta \;=\;\left(1-f\right)D_{e}^{⊥},\gamma \;=\;fD_{a}^{2}+\left(1-f\right)\Delta_{e}^{2},\\ \delta \; & =\;\left(1-f\right)\Delta_{e}D_{e}^{⊥},\epsilon \;=\;\left(1-f\right)D_{e}^{⊥2}. \end{array}$$

After this substitution, Equation 6 can be expressed as a linear system of five equations for the 5 unknowns *α*,* β*,* γ*,* δ* and *ε*, decoupled into two independent smaller systems:

(8) $$\begin{array}{cc} \left[\begin{array}{c} D_{\|}\\ D_{⊥} \end{array}\right]\; & =\;\left[\begin{array}{cc} h_{2}\left(1,\kappa \right) & 1\\ h_{2}\left(0,\kappa \right) & 1 \end{array}\right]\left[\begin{array}{c} \alpha \\ \beta \end{array}\right]\;=\;L\left[\begin{array}{c} \alpha \\ \beta \end{array}\right],\\ \left[\begin{array}{c} \frac{1}{3}W_{\|}{\overset{¯}{D}}^{2}+D_{\|}^{2}\\ \frac{1}{3}W_{⊥}{\overset{¯}{D}}^{2}+D_{⊥}^{2}\\ \frac{5\overset{¯}{W}{\overset{¯}{D}}^{2}}{8}-\frac{W_{⊥}{\overset{¯}{D}}^{2}}{4}-\frac{W_{\|}{\overset{¯}{D}}^{2}}{24}+\frac{{\left(D_{⊥}+D_{\|}\right)}^{2}}{4} \end{array}\right]\; & =\;\left[\begin{array}{ccc} h_{4}\left(1,\kappa \right) & 2h_{2}\left(1,\kappa \right) & 1\\ h_{4}\left(0,\kappa \right) & 2h_{2}\left(0,\kappa \right) & 1\\ h_{4}\left(\frac{1}{\sqrt{2}},\kappa \right) & 2h_{2}\left(\frac{1}{\sqrt{2}},\kappa \right) & 1 \end{array}\right]\left[\begin{array}{c} \gamma \\ \delta \\ \epsilon \end{array}\right]\;=\;M\left[\begin{array}{c} \gamma \\ \delta \\ \epsilon \end{array}\right]. \end{array}$$

**Table 1:** Illustration of sets of biophysical (BP) parameter values resulting in the same diffusion–kurtosis (DK) parameters

DK parameters		BP parameters		*C* new invariants
$\left[D_{\|},\;D_{⊥},\;W_{\|},\;W_{⊥},\;\overset{¯}{W}\right]$	Branch	$\left[f,\;D_{a},\;D_{e}^{\|},\;D_{e}^{⊥},\;\kappa \right]$	**ζ**_**1**_	**ζ**_**2**_
[1.503, 0.195, 1.456, 0.291, 0.926]	+	[0.730, 2.000, 1.000, 0.300, 8.000]	−0.006	0.210
	−	[0.607, 1.287, 2.191, 0.318, 11.49]	0.023	0.053
[1.557, 1.048, 0.396, 0.708, 0.330]	+	[0.250, 2.370, 1.300, 1.390, 50.00]	0.349	0.624
	−	—		—
[0.457, 0.408, 2.901, 2.702, 2.770]	+	[0.879, 1.320, 1.401, −0.232, 0.265]	−0.190	0.022
	−	[0.870, 0.950, 2.000, 0.720, 0.360]	−0.023	0.014
	−	[0.549, 0.182, 1.071, 0.766, 1.414]	0.154	−0.002
	−	[0.510, 0.076, 0.931, 0.794, 3.187]	0.161	−0.005
[1.560, 1.256, 0.423, 0.540, 0.506]	+	—		—
	−	[0.240, 1.450, 2.100, 1.400, 2.330]	0.237	0.125
	−	[0.189, 0.668, 1.887, 1.489, 5.442]	0.325	0.057

Each plus or minus branch can correspond to a single, multiple, or none BP parameters. Some sets of BP parameters fall outside the region of plausible parameters, like the + branch solution of the third example. We can observe that the invariants of the not fully symmetric part of **C**, incorporated by DDE, discriminate between the BP parameter sets having the same exact DK representation. All diffusivities are in $\mu {\text{m}}^{2}/\text{ms}$ and the *C* components in $\mu {\text{m}}^{4}/\;{\text{ms}}^{2}$.

Observe that the coefficients of matrices **L** and **M** depend on *κ*. We will ignore for the moment that the five unknowns are not independent. The solution is unique as long as matrices **L** and **M** are invertible. This is the case when *κ* ≠ 0, since $\det L\;=\;p_{2}$ and $\det M\;=\;-\frac{1}{2}p_{2}p_{4}$. In the limit of a fully isotropic medium (*κ* = 0) the system has only two independent equations, not allowing the recovering of the kernel parameters without additional information. By solving the two systems in Equation 8 we find expressions for *α*,* β*,* γ*,* δ* and *ε* that only depend on *κ* and the DK parameters (see Appendix A for solution). Those variables are actually defined from only four kernel parameters (Equation 7), resulting in the coupling equation

(9) $$\gamma \left(\epsilon -\beta^{2}\right)\;=\;\alpha^{2}\epsilon +\delta^{2}-2\alpha \beta \delta .$$

By plugging the expressions for *α*,* β*,* γ*,* δ* and *ε* as functions of *κ* into Equation 9, we obtain a nonlinear equation for *κ* with potentially multiple solutions. Each solution for *κ* gives a single solution for *α*,* β*,* γ*,* δ* and *ε*, which in turn, gives a single solution for the kernel parameters:

(10) $$f\;=\;1-\frac{\beta^{2}}{\epsilon },\;D_{a}\;=\;\frac{\alpha \epsilon -\beta \delta }{\epsilon -\beta^{2}},\;\Delta_{e}\;=\;\frac{\delta }{\beta },\;D_{e}^{⊥}\;=\;\frac{\epsilon }{\beta }.$$

Thus, the number of solutions to Equation 9 corresponds to the number of BP parameter sets that have the same DK parameters. Table 1 presents cases with up to four solutions. We computed the number of solutions for 10k random points in the BP parameter space. Most present two solutions (70.2%), some only one (29.3%), and only a small proportion have four solutions (0.5%). This gives rise to the previously discussed degeneracy in model parameter estimation from noisy measurements.^17^ In contrast with the claim in Hansen et al^51^ even in the extreme case of parallel fibers leaving only four unknowns, the five equations in Equation 6 are independent. This is possible due to the nonlinear nature of the system. If *κ* is known and not zero (including the limiting case *κ* → ∞ of parallel fibers), the full‐system is invertible as long as *f* is not 0 or 1, and $D_{e}^{⊥}$ is not null. In that case, each point in the DK parameter space (signal profile) corresponds to a single set of BP parameters. However, this is not the case for an arbitrary unknown *κ*. Here, the full‐system has five independent equations with five unknowns, but, depending on the parameter values, it can have only one or multiple solutions. This latter case makes the inverse mapping an ill‐posed problem.

Using very high b‐values might be considered an option to solve this problem, as it will add higher order terms in Equation 3. However, it is still challenging due to very low associated signal‐to‐noise ratio (SNR) and is also unfeasible in most clinical scanners, although bespoke systems with ultra‐strong gradients may provide leverage in this regard.^52^ Another solution that does not require powerful gradients is to seek for independent measurements providing new information.

### 2.4 Model extension to DDE

DDE adds an extra dimension to the dMRI acquisition, unexplored by SDE experiments. For a general multidimensional acquisition,^32^, ^53^ due to the assumption of impermeable compartments, within each of which the diffusion displacement profile is assumed to be Gaussian, the signal can be written as:

(11) $$S_{\mathrm{NODDIDA}}\left(B\right)=S_{0}\int_{S^{2}}P\left(\hat{u}\right)K\left(B,\hat{u}\right)dS_{\hat{u}},$$

with the kernel

(12) $$K\left(B,\hat{u}\right)\;=\;f\exp\left[-D_{a}B_{\mathrm{ij}}u_{i}u_{j}\right]+\left(1-f\right)\exp\left[-bD_{e}^{⊥}-\Delta_{e}B_{\mathrm{ij}}u_{i}u_{j}\right],$$

for *b* = *tr*(**B**). The b‐tensor of a DDE acquisition is $B\;=\;b_{1}{\hat{n}}_{1}\;⊗\;{\hat{n}}_{1}\;+\;b_{2}{\hat{n}}_{2}\;⊗\;{\hat{n}}_{2}$, defined from the pair of gradient directions, ${\hat{n}}_{1}$, ${\hat{n}}_{2}$, and their individual diffusion weightings, $b_{1}$, $b_{2}$. It has in general two non‐zero eigenvalues, *viz*. PTE. In contrast, the SDE’s b‐tensor, $B\;=\;b\hat{n}\;⊗\;\hat{n}$, has only one non‐zero eigenvalue, *viz*. LTE. Hence, for this model a SDE acquisition is a subset of the DDE acquisitions ($\text{SDE}\;=\;{\text{DDE}}_{\|}\;\subset \;\text{DDE}$), for which ${\hat{n}}_{1}\;=\;{\hat{n}}_{2}$ (parallel direction pair).

### 2.5 DDE information gain

DDE can, in principle, provide independent complementary information. This could transform the inverse mapping of recovering BP parameters from diffusion‐weighted measurements into a well‐posed problem. The fourth order cumulant expansion for the dMRI signal arising from a DDE experiment is

(13) $$\begin{array}{cc} \log(S/S_{0}) & \;=\;-B_{\mathrm{ij}}D_{\mathrm{ij}}\;+\;\frac{1}{2}B_{\mathrm{ij}}B_{k\ell }C_{ijk\ell }\\ & \;=\;-\left(b_{1}n_{1i}n_{1j}\;+\;b_{2}n_{2i}n_{2j}\right)D_{\mathrm{ij}}\;+\;\frac{{\overset{¯}{D}}^{2}}{6}\left(b_{1}^{2}n_{1i}n_{1j}n_{1k}n_{1\ell }\;+\;b_{2}^{2}n_{2i}n_{2j}n_{2k}n_{2\ell }\right)W_{ijk\ell }\;+\;b_{1}b_{2}n_{1i}n_{1j}n_{2k}n_{2\ell }C_{ijk\ell }. \end{array}$$

Here, **C** is the second cumulant tensor of the dMRI signal expansion in terms of the b‐tensor and satisfies minor and major symmetries:

(14) $$C_{ijk\ell }\;=\;C_{jik\ell }\;=\;C_{ij\ell k}\;=\;C_{k\ell ij},$$

but it is not totally symmetric. Its totally symmetric part is proportional to the kurtosis tensor:

(15) $${\overset{¯}{D}}^{2}W_{ijk\ell }\;=\;3C_{\left(ijk\ell \right)}\;=\;C_{ijk\ell }\;+\;C_{i\ell jk}\;+\;C_{ik\ell j}.$$

For MGCs or DDE with long mixing times,^31^ **D** and **C** can be written as

(16) $$\begin{array}{cc} D_{\mathrm{ij}} & \;=\;〈D_{\mathrm{ij}}〉\;=\;\sum_{\alpha }f_{\alpha }D_{\mathrm{ij}}^{\left(\alpha \right)},\\ C_{ijk\ell } & \;=\;〈\left(D_{\mathrm{ij}}-\left\langle D_{\mathrm{ij}}\right\rangle \right)\left(D_{k\ell }-\left\langle D_{k\ell }\right\rangle \right)〉\\ \; & =\;\sum_{\alpha }f_{\alpha }D_{\mathrm{ij}}^{\left(\alpha \right)}D_{k\ell }^{\left(\alpha \right)}-D_{\mathrm{ij}}D_{k\ell }, \end{array}$$

where $f_{\alpha }$ and $D_{\mathrm{ij}}^{\left(\alpha \right)}$ denote the fraction and diffusion tensor of compartment *α*, including in this summation the integral over the unit sphere with the ODF (cf. Equation 1). This motivated naming **C** as the diffusion tensor covariance.^31^, ^32^ Our definition of **C** coincides with the one in Westin et al^32^ and for long mixing times it is also proportional to the **Z** tensor ($C\;=\;Z/(4\Delta^{2})$), earlier introduced in Jespersen.^31^ The **Z** tensor is defined more generally, i.e. not restricted to MGCs, as a cumulant of the DDE signal.

In the case of a Watson ODF, **W** and **C** are transversely isotropic fourth order tensors, i.e. they have cylindrical symmetry. Hence, instead of having 15 and 21 independent components they only have three and five, respectively. We can write both tensors as a function of coordinate independent tensor forms (for full derivation see Appendix B), like it is done for **W** in Hansen et al^51^ (Equation 6):

(17) $$W\;=\;\omega_{1}P\;+\;\omega_{2}Q\;+\;\omega_{3}I\;\text{and}\;C\;=\;\frac{1}{3}{\overset{¯}{D}}^{2}W\;+\;\zeta_{1}R\;+\;\zeta_{2}J,$$

where **C** was written separating its fully symmetric part from the remaining part,^54^ and

(18) $$\begin{array}{cc} P_{ijk\ell } & \;=\;\mu_{i}\mu_{j}\mu_{k}\mu_{\ell },\\ Q_{ijk\ell } & \;=\;\frac{1}{6}\left(\mu_{i}\mu_{j}\delta_{k\ell }\;+\;\mu_{k}\mu_{\ell }\delta_{\mathrm{ij}}\;+\;\mu_{i}\mu_{k}\delta_{j\ell }\;+\;\mu_{j}\mu_{k}\delta_{i\ell }\;+\;\mu_{i}\mu_{\ell }\delta_{\mathrm{jk}}\;+\;\mu_{j}\mu_{\ell }\delta_{\mathrm{ik}}\right),\\ I_{ijk\ell } & \;=\;\frac{1}{3}\left(\delta_{\mathrm{ij}}\delta_{k\ell }\;+\;\delta_{\mathrm{ik}}\delta_{j\ell }\;+\;\delta_{i\ell }\delta_{\mathrm{jk}}\right),\\ R_{ijk\ell } & \;=\;\frac{1}{2}\left(\mu_{i}\mu_{j}\delta_{k\ell }\;+\;\mu_{k}\mu_{\ell }\delta_{\mathrm{ij}}\right)-\frac{1}{4}\left(\mu_{i}\mu_{k}\delta_{j\ell }\;+\;\mu_{j}\mu_{k}\delta_{i\ell }\;+\;\mu_{i}\mu_{\ell }\delta_{\mathrm{jk}}\;+\;\mu_{j}\mu_{\ell }\delta_{\mathrm{ik}}\right),\\ J_{ijk\ell } & \;=\;\delta_{\mathrm{ij}}\delta_{k\ell }-\frac{1}{2}\left(\delta_{\mathrm{ik}}\delta_{j\ell }\;+\;\delta_{i\ell }\delta_{\mathrm{jk}}\right), \end{array}$$

where $\delta_{\mathrm{ij}}$ is the Kronecker delta and $\hat{\mu }$ the Watson distribution main direction. Equation 17 shows explicitly that **C** contains two extra degrees of freedom independent of **W**. Observe that the fully symmetric part of **R** and **J** vanishes, so that the information encoded in $\zeta_{1}$ and $\zeta_{2}$ is not accessible from a SDE experiment.^28^ We can isolate the new non‐symmetric components by the antisymmetrization

(19) $$C_{ijk\ell }-C_{ikj\ell }\;=\;\zeta_{1}\left(R_{ijk\ell }-R_{ikj\ell }\right)\;+\;\zeta_{2}\left(J_{ijk\ell }-J_{ikj\ell }\right).$$

Considering a coordinate frame with the *z*‐axis parallel to the fibers main direction $\hat{\mu }$, we can identify

(20) $$C_{\mathrm{xxyy}}-C_{\mathrm{xyxy}}\;=\;\frac{3}{2}\zeta_{2}\;\text{and}\;C_{\mathrm{xxzz}}-C_{\mathrm{xzxz}}-C_{\mathrm{xxyy}}\;+\;C_{\mathrm{xyxy}}\;=\;\frac{3}{4}\zeta_{1}.$$

Similarly to Equation 6 we can relate the elements of **C** to the biophysical parameters like it was done for **W**. For the SM, including NODDIDA, **D** and **C** are given by

(21) $$\begin{array}{cc} D_{\mathrm{ij}} & \;=\;\left[fD_{a}\;+\;\left(1-f\right)\Delta_{e}\right]H_{\mathrm{ij}}^{\left(2\right)}\;+\;\left(1-f\right)D_{e}^{⊥}\delta_{\mathrm{ij}},\\ C_{ijk\ell } & \;=\;\left[fD_{a}^{2}\;+\;\left(1-f\right)\Delta_{e}^{2}\right]H_{ijk\ell }^{\left(4\right)}\;\\ +\;\left(1-f\right)D_{e}^{⊥}\Delta_{e}\left(\delta_{\mathrm{ij}}H_{k\ell }^{\left(2\right)}\;+\;\delta_{k\ell }H_{\mathrm{ij}}^{\left(2\right)}\right)\;\\ +\;\left(1-f\right)D_{e}^{⊥2}\delta_{\mathrm{ij}}\delta_{k\ell }-D_{\mathrm{ij}}D_{k\ell }, \end{array}$$

where

(22) $$H_{\mathrm{ij}}^{\left(2\right)}\;=\;\int_{S^{2}}P\left(\hat{u}\right)u_{i}u_{j}dS_{\hat{u}}\;\text{and}\;H_{ijk\ell }^{\left(4\right)}\;=\;\int_{S^{2}}P\left(\hat{u}\right)u_{i}u_{j}u_{k}u_{\ell }dS_{\hat{u}}.$$

For NODDIDA we get $h_{2}\left(\xi ,\;\kappa \right)\;=\;H_{\mathrm{ij}}^{\left(2\right)}n_{i}n_{j}$ and $h_{4}\left(\xi ,\;\kappa \right)\;=\;H_{ijk\ell }^{\left(4\right)}n_{i}n_{j}n_{k}n_{\ell }$, with $\xi \;=\;\hat{\mu }\cdot \hat{n}$. The cross‐terms of **C** present new information not accessible from SDE. This makes the DDE signal able to resolve the degeneracy. To make this explicit, we can write the components isolated in Equation 20 in the adapted coordinate frame in terms of BP parameters:

(23) $$\begin{array}{cc} \frac{3}{2}\zeta_{2} & \;=\;C_{\mathrm{xxyy}}-C_{\mathrm{xyxy}}\;=\;\left(1-f\right)\left[D_{e}^{⊥}\Delta_{e}\left(H_{\mathrm{xx}}^{\left(2\right)}\;+\;H_{\mathrm{yy}}^{\left(2\right)}\right)\;+\;{D_{e}^{⊥}}^{2}\right]-D_{\mathrm{xx}}D_{\mathrm{yy}}\\ & \;=\;\left(1-f\right)\left[2D_{e}^{⊥}\Delta_{e}h_{2}\left(0,\kappa \right)\;+\;{D_{e}^{⊥}}^{2}\right]-D_{⊥}^{2},\\ \frac{3}{4}\zeta_{1} & \;=\;C_{\mathrm{xxzz}}-C_{\mathrm{xzxz}}-C_{\mathrm{xxyy}}\;+\;C_{\mathrm{xyxy}}\;=\;\left(1-f\right)D_{e}^{⊥}\Delta_{e}\left(H_{\mathrm{zz}}^{\left(2\right)}-H_{\mathrm{yy}}^{\left(2\right)}\right)-D_{\mathrm{xx}}\left(D_{\mathrm{zz}}-D_{\mathrm{yy}}\right)\\ & \;=\;\left(1-f\right)D_{e}^{⊥}\Delta_{e}\left(h_{2}\left(1,\kappa \right)-h_{2}\left(0,\kappa \right)\right)-D_{⊥}\left(D_{\|}-D_{⊥}\right) \end{array}.$$

Those two equations are independent to the ones in Equation 6, adding complementary information to the mapping between DK and BP spaces (see last column in Table 1). Using the same variables defined in Equation 7 we get

(24) $$2h_{2}\left(0,\kappa \right)\delta \;+\;\epsilon \;=\;\frac{3}{2}\zeta_{2}\;+\;D_{⊥}^{2}\;\text{and}\;\left(h_{2}\left(1,\kappa \right)-h_{2}\left(0,\kappa \right)\right)\delta \;=\;\frac{3}{4}\zeta_{1}\;+\;D_{⊥}\left(D_{\|}-D_{⊥}\right).$$

These two equations enlarge the system in Equation 8. Following the derivation in Appendix C, we demonstrate that they determine a single solution for *κ*:

(25) $$\frac{h_{4}\left(1,\kappa \right)}{h_{4}\left(0,\kappa \right)}\;=\;\frac{\frac{1}{3}W_{\|}{\overset{¯}{D}}^{2}-\frac{3}{2}\left(\zeta_{1}\;+\;\zeta_{2}\right)\;+\;{\left(D_{\|}-D_{⊥}\right)}^{2}}{\frac{1}{3}W_{⊥}{\overset{¯}{D}}^{2}-\frac{3}{2}\zeta_{2}}$$

since the left‐hand side is a strictly monotone increasing function on *κ*. This agrees with recent work by Cotaar et al,^55^ who showed that combining different b‐tensor shapes can determine robustly fiber dispersion. Observe that the cases *f* = 0 or *f* = 1 reflect only an apparent degeneracy, as the different sets of parameters represent the same physical model. In contrast, the case of *κ* = 0 presents a proper degeneracy of the model due to lack of information, where different model instances have identical **D** and **C** tensors.

## 3 METHODS

### 3.1 Signal generation

All synthetic measurements were generated from substrates composed of 1 μm diameter cylinders to simultaneously assess our stick approximation. We found this difference was below the noise level. We computed the signal attenuation in the cylinder’s perpendicular plane with the Gaussian phase approximation for both SDE^56^ and DDE.^30^

Since there is no closed analytical solution for the integral on the sphere in Equation 11, we computed the spherical convolution using Lebedev’s quadrature^57^:

(26) $$\int_{S^{2}}f\left(\hat{u}\right)dS_{\hat{u}}\approx \sum_{i}w_{i}f\left({\hat{u}}_{i}\right),$$

where $w_{i}$ are the quadrature weights of each grid point ${\hat{u}}_{i}$ across the unit sphere. For all configurations of SDE and DDE we used 1,202 quadrature points, which guarantee an exact result up to a $59^{\mathrm{th}}$ order spherical harmonics decomposition of the ODF. No practical differences were found between the results from our SDE implementation and the analytic summation for SDE in Zhang et al.^43^

Finally, Rician noise was added to the synthetic signals, normalizing it to obtain a SNR = 50 for the $b_{0}$ measurements, like in Jelescu et al.^17^

### 3.2 Parameter estimation algorithm

Parameter estimation was based on a nonlinear least squares estimator. This was justified due to the relatively high SNR considered for the experiments, where Rician noise can be approximated as Gaussian.^58^ We used the Trust Region Reflective algorithm implemented in the MATLAB (R2016a, MathWorks, Natick, Massachusetts) optimization toolbox. The objective cost function was

(27) $$F\left(\theta \right)\;=\;\sum_{i}^{N}{\left(S\left(B_{i},\theta \right)-S_{\mathrm{NODDIDA}}\left(B_{i},\theta \right)\right)}^{2},$$

where N is the total number of measurements, $B_{i}$ indicates the b‐tensor used in the *i*‐th measurement and $\theta \;=\;[f,D_{a},\;D_{e}^{\|},\;D_{e}^{⊥},\;\kappa ]$ is a vector containing the model parameters. The main direction of the fibers, $\hat{\mu }$, and $S_{0}$ were fitted independently in a first stage through a DTI fitting like in Jelescu et al.^17^ For all configurations, this optimization procedure was repeated using 30 independent random initializations for the model parameters to avoid local minima of the five‐dimensional cost function. The local solution with the lowest residue was the global optimum.

### 3.3 SDE and DDE tested configurations

**Figure 2:**
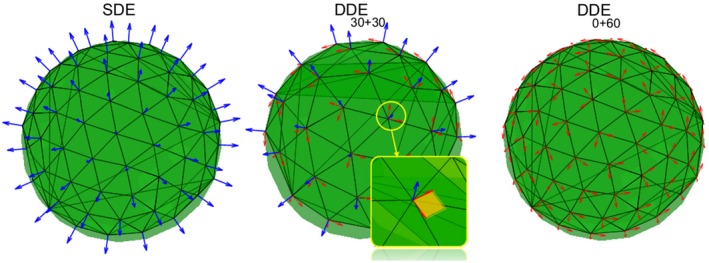
Diagram of different measurement protocols (SDE, ${\text{DDE}}_{30\;+\;30}$, and ${\text{DDE}}_{0\;+\;60}$). Only SDE and ${\text{DDE}}_{30\;+\;30}$ were used in experiment 1, while they all were used in experiment 2. Blue colors denote the SDE directions or DDE parallel direction pairs. DDE perpendicular direction pairs are in red

Five encoding configurations were considered: ${\text{DDE}}_{60\;+\;0}$, ${\text{DDE}}_{40\;+\;20}$, ${\text{DDE}}_{30\;+\;30}$, ${\text{DDE}}_{20\;+\;40}$, and ${\text{DDE}}_{0\;+\;60}$, with progressively increasing proportions of perpendicular direction pairs, *b*, with respect to parallel direction pairs, *a*, denoted as ${\text{DDE}}_{a\;+\;b}$. Observe that ${\text{DDE}}_{60\;+\;0}$ is equivalent to SDE if MGCs are assumed. We compared the SDE protocol used in Jelescu et al^17^ against different DDE acquisitions with the same number of measurements that can be measured in a similar experimental time. The SDE measurement protocol had two shells with b‐values of 1 and $2\;\text{ms}/\mu {\text{m}}^{2}$ with 30 directions each.^17^ These directions were generated using the Sparse and Optimal Acquisition (SOA) scheme.^59^ DDE configurations were also divided in two shells with the same b‐values as above and both directions in each pair had equal individual diffusion weightings, $b_{1}\;=\;b_{2}\;=\;\frac{1}{2}b$. Thus, perpendicular direction pairs define axially symmetric planar b‐tensors, uniquely defined by their normal vector. We generated homogeneously distributed normal vectors using the same algorithm used for the SDE directions. The ${\text{DDE}}_{30\;+\;30}$ acquisition had 30 parallel direction pairs and 30 perpendicular direction pairs with normal vectors coinciding with the parallel pairs^33^ (see middle diagram in Figure 2). The ${\text{DDE}}_{0\;+\;60}$ protocol had only perpendicular directions pairs (right diagram in Figure 2). Configuration ${\text{DDE}}_{40\;+\;20}$ had two parallel per each perpendicular directions pair, and ${\text{DDE}}_{20\;+\;40}$ two perpendicular per each parallel directions pair. All acquisitions had five non diffusion‐weighted measurements (i.e. $b_{0}$ measurements).

### 3.4 Experiments

We performed two *in silico* experiments to assess whether the addition of DDE measurements can enhance the parameter estimation in the presence of typical noise in the measurements.

In the first experiment, we considered two possible instances of NODDIDA parameter values for a voxel in the posterior limb of the internal capsule (PLIC) taken from Jelescu et al^17^ (see Table 2), for which SDE estimates showed a bimodal distribution. We explored in detail whether DDE solves the degeneracy between these particular cases. Only SDE and ${\text{DDE}}_{30\;+\;30}$ acquisition configurations were considered for this experiment. Two thousand and five hundred independent realizations of Rician noise were added to the synthetic SDE and DDE signals.

**Table 2:** Ground truth for experiment 1

Model parameter	SET A	SET B
f	0.38	0.77
${\text{D}}_{a}\left[\mu {\text{m}}^{2}/\text{ms}\right]$	0.50	2.23
${\text{D}}_{e}^{\|}\left[\mu {\text{m}}^{2}/\text{ms}\right]$	2.10	0.16
${\text{D}}_{e}^{⊥}\left[\mu {\text{m}}^{2}/\text{ms}\right]$	0.74	1.48
${\text{c}}_{2}\left(\kappa \right)$	0.98 (64)	0.70 (4)

The second experiment aims to compare the accuracy and precision provided by SDE and the different DDE configurations extensively along the feasible region of the full five‐dimensional (5D) space of parameters (diffusivities between 0 and $3\;\mu {\text{m}}^{2}/\text{ms}$, fraction between 0 and 1, and *κ* positive). This allows exploring whether there are subregions presenting different behaviors. A 5D grid was generated by all the combinations of *f* = [0.1, 0.3, 0.5, 0.7, 0.9], $D_{a}\;=\;\left[0.3,0.8,1.3,1.8,2.3\right]\mu {\text{m}}^{2}/\text{ms}$, $D_{e}^{\|}\;=\;\left[0.8,\;1.3,\;1.8\right]\mu {\text{m}}^{2}/\text{ms}$, $D_{e}^{⊥}\;=\;\left[0.5,\;1,\;1.5\right]\mu {\text{m}}^{2}/\text{ms}$, and *κ* = [0.84, 2.58, 4.75, 9.27, 15.53, 33.70]. The fraction and the diffusivities were selected from a uniform discretization of the expected range, and *κ* values were chosen such that the mean‐squared‐cosine corresponding angle, $\left\langle \cos^{2}\varphi \right\rangle \;=\;c_{2}\;=\;\left\langle {\left(\hat{u}\cdot \hat{\mu }\right)}^{2}\right\rangle \;=\;{\left(2\sqrt{\kappa }F\left(\sqrt{\kappa }\right)\right)}^{-1}-{\left(2\kappa \right)}^{-1}$, was $\varphi \;=\;[50^{\circ },\;40^{\circ },\;30^{\circ },\;20^{\circ },\;15^{\circ },\;10^{\circ }]$ ($c_{2}\;=$ [0.41, 0.59, 0.75, 0.88, 0.93, 0.97]). We generated 50 independent Rician noise realizations (SNR = 50) for the measurements of each combination of the parameters for the five configurations.

## 4 RESULTS

**Figure 3:**
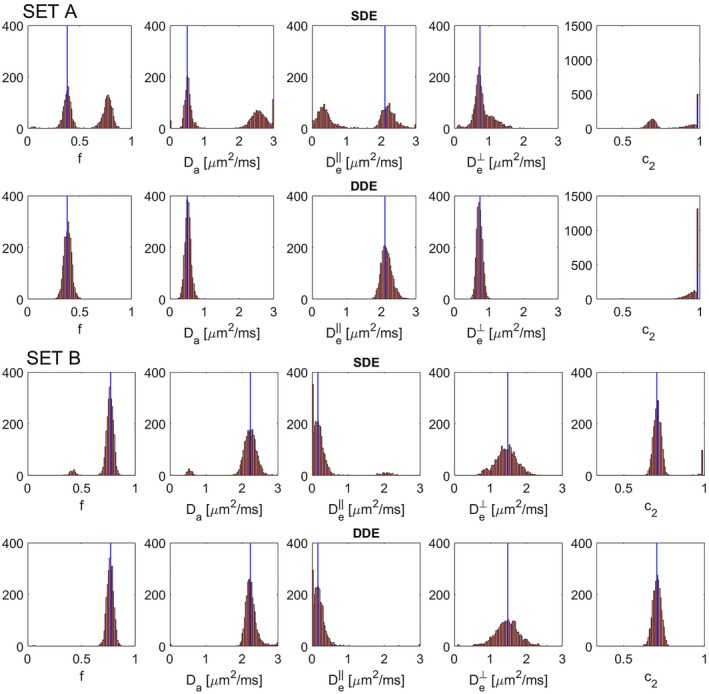
Histograms of the estimated model parameters for SDE (top row) and ${\text{DDE}}_{30\;+\;30}$ (bottom row) schemes in the first experiment for 2,500 independent noise realizations (SNR = 50). The ground truth represents two possible solutions of the NODDIDA model applied to a voxel in the PLIC (Table 2). These values are shown in blue lines and correspond to set A (upper two rows), and set B (lower two rows)

Histograms of the estimated model parameters from the first experiment (Figure 3) show an increase in the accuracy and precision of the estimates with the DDE scheme. The bimodal distribution of the estimated parameters is evident with the SDE acquisition, confirming that it is not possible to differentiate true and spurious minima. This effect is removed when using the DDE sequence.

**Figure 4:**
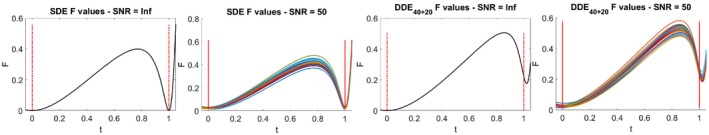
Plots of ${\text{F}}_{A}\left(\theta \left(t\right)\right)$ for different values of *t* ∈ [−0.05,1.05], with $\theta \left(t\right)\;=\;t\theta_{\mathrm{spur}}\;+\;\left(1-t\right)\theta_{\mathrm{true}}$. Black curves show ${\text{F}}_{A}$ values for noise‐free SDE and ${\text{DDE}}_{30\;+\;30}$ acquisitions. The colored curves show 30 independent realizations of ${\text{F}}_{A}$ for SNR = 50

We analyzed the shapes of the SDE and DDE objective functions from the synthetic measurements of SET A (sum of squared differences: ${\text{F}}_{A}\left(\theta \right)$). To facilitate the visualization of these 5D functions, we performed a 1D cut through a straight line joining the true and spurious minima of SDE. This was parametrized with the scalar variable *t*: $\theta \;=\;t\theta_{\mathrm{spur}}\;+\;\left(1-t\right)\theta_{\mathrm{true}};\;t\;\in \;\left[0,\;1\right]$, where $\theta_{\mathrm{true}}\;=\;\left[0.38,\;0.5,\;2.1,\;0.74,\;64\right]$ and $\theta_{\mathrm{spur}}\;=\;\left[0.78,\;2.67,\;0.32,\;0.85,\;3.65\right]$, with diffusivities expressed in $\mu {\text{m}}^{2}/\text{ms}$. Figure 4 shows the behavior of ${\text{F}}_{A}\left(\theta \right)$ along this cut as a function of *t*. It can be observed that although the DDE objective function is still bimodal, the spurious and true minima have significantly different absolute values (due to the contribution of the tensor **C** to the DDE signal). This enables us to distinguish both peaks in conditions where SDE cannot (i.e. ${\text{b}}_{\max}\;=\;2\;\text{ms}/\mu {\text{m}}^{2}$). Adding more directions to the SDE acquisition would not help to differentiate the peaks, as even in the noiseless case these two sets produce the same signal. Only by increasing the SDE diffusion weighting the spurious minimum could be differentiated from the true one.

**Figure 5:**
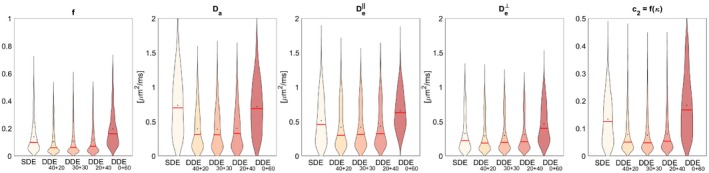
Violin plots of the RMSE for all model parameters for all voxels in the 5D grid (a total of 5×5×3×3×6 = 1,350). Black dots denote the mean and red lines the median. The RMSE for each voxel was computed by repeating the estimation on 50 independent noise realizations of the measurements for each voxel

For each point in the 5D grid of parameters, the Root Mean Square Error (RMSE, for definition see for instance^60^) of each parameter has been computed from 50 independent noise realizations. The distributions of RMSE of the parameter estimates from this second experiment are displayed in Figure 5 with violin plots (similar to box plots but showing also estimated probability density^61^). The summary statistics of the RMSE distributions are shown in Table 3. On average, ${\text{DDE}}_{40\;+\;20}$ and ${\text{DDE}}_{30\;+\;30}$ are the most accurate configurations for estimating all parameters. This suggests that the incorporation of even a small proportion of DDE measurements can remove the degeneracy, leading to an increase in accuracy and precision.

**Table 3:** Mean and standard deviation of the RMSE over the whole grid for each acquisition protocol and each of the estimated parameters

RMSE (*μ*; *σ*)	*f*	$D_{a}\left[\mu {\text{m}}^{2}/\text{ms}\right]$	$D_{e}^{\|}\left[\mu {\text{m}}^{2}/\text{ms}\right]$	$D_{e}^{⊥}\left[\mu {\text{m}}^{2}/\text{ms}\right]$	$c_{2}\;=\;f\left(\kappa \right)$
SDE	0.14; 0.12	0.74; 0.43	0.51; 0.33	0.33; 0.27	0.13; 0.08
${\text{DDE}}_{40\;+\;20}$	0.10; 0.10	0.39; 0.30	0.41; 0.31	0.29; 0.25	0.08; 0.07
${\text{DDE}}_{30\;+\;30}$	0.11; 0.10	0.39; 0.29	0.42; 0.30	0.30; 0.25	0.08; 0.07
${\text{DDE}}_{20\;+\;40}$	0.11; 0.10	0.40; 0.30	0.43; 0.31	0.31; 0.25	0.08 ; 0.07
${\text{DDE}}_{0\;+\;60}$	0.20; 0.13	0.72; 0.38	0.65; 0.28	0.46; 0.27	0.18; 0.11

**Figure 6:**
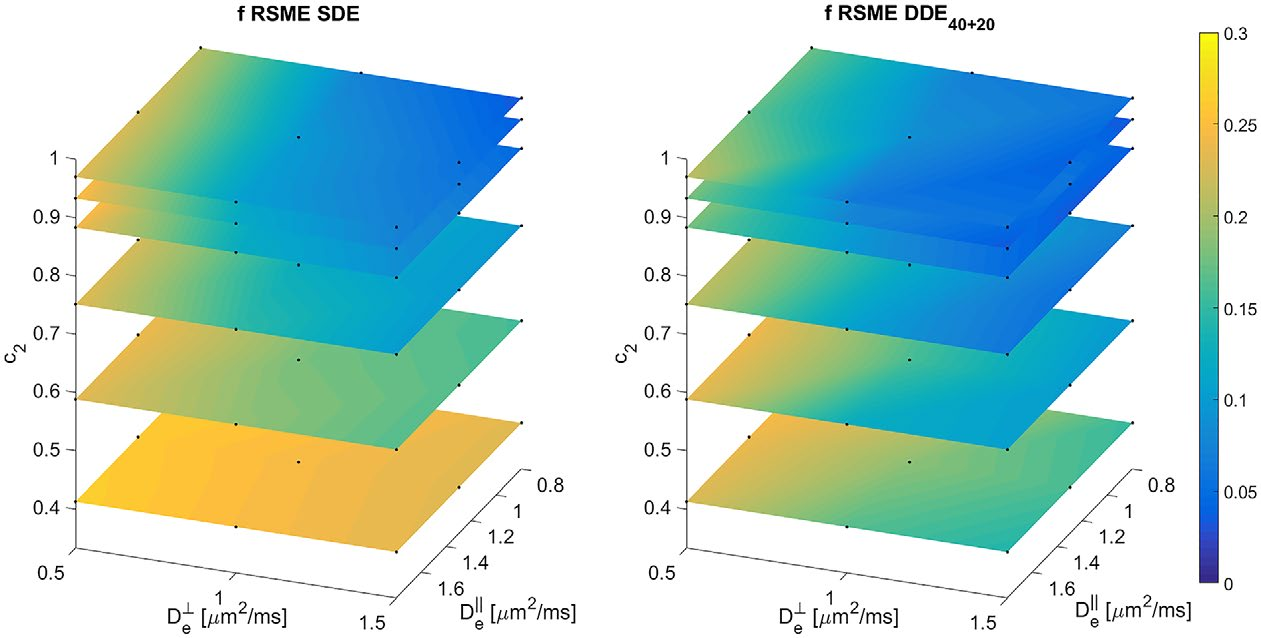
RMSE of *f*, for SDE and ${\text{DDE}}_{40\;+\;20}$ acquisition protocols. This 3D plot shows the projection over $D_{e}^{\|}$, $D_{e}^{⊥}$, and $c_{2}$ of all the RMSE in the 5D grid. This projection was made by computing the square root of the quadratic mean of the errors in the remaining 2 dimensions (${\text{E}}_{proj,ijk}\;=\;\sqrt{\sum_{\ell }\sum_{m}E_{ijk\ell m}^{2}/\left(N_{\ell }N_{m}\right)}$). Black dots denote the actual points in the grid, linear interpolation was used to generate the color figures

**Figure 7:**
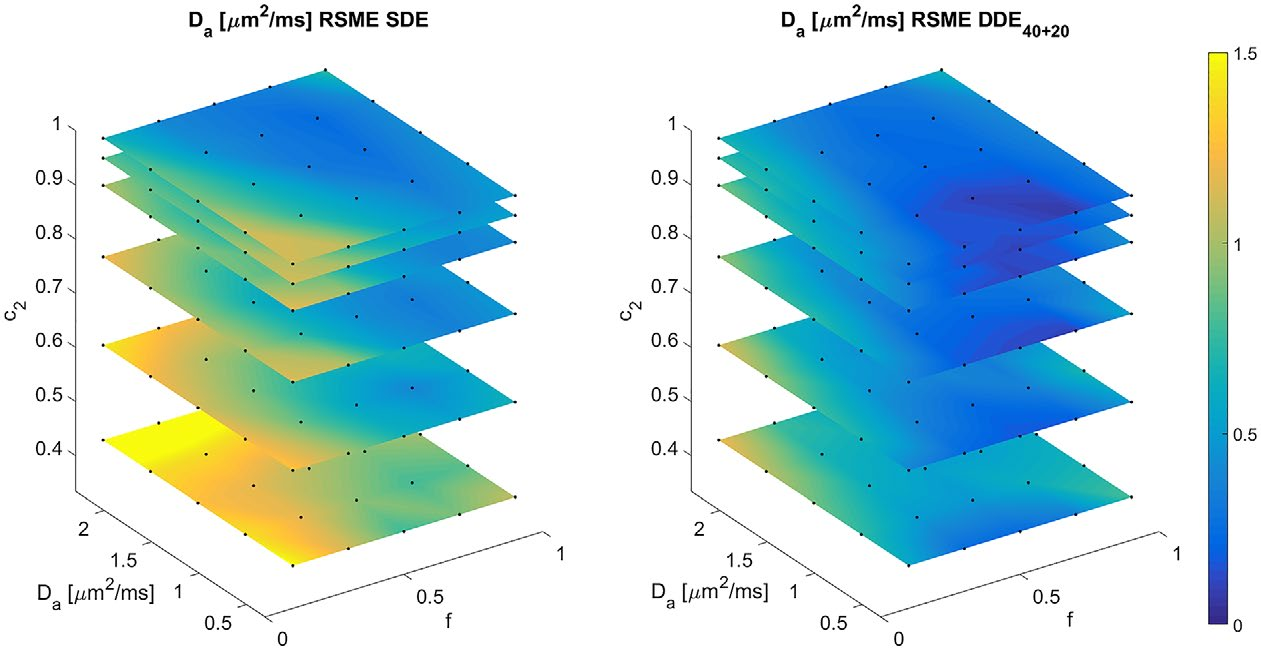
RMSE of $D_{a}$, for SDE and ${\text{DDE}}_{40\;+\;20}$ acquisition protocols. This 3D plot shows the projection over *f*, $D_{a}$, and $c_{2}$ of all the RMSE in the 5D grid. This projection was made by computing the square root of the quadratic mean of the errors in the remaining 2 dimensions (${\text{E}}_{proj,ijk}\;=\;\sqrt{\sum_{\ell }\sum_{m}E_{ijk\ell m}^{2}/\left(N_{\ell }N_{m}\right)}$). Black dots denote the actual points in the grid, linear interpolation was used to generate the color figures

To compare the performance of SDE and DDE in different regions of the parameter space, we projected the 5D RMSE map onto different 3D sub‐spaces. Figures 6 and 7 show two different 3D projections, over $\left({\text{D}}_{e}^{\|},\;{\text{D}}_{e}^{⊥},\;c_{2}\left(\kappa \right)\right)$ and over $\left(f,{\text{D}}_{a},\;c_{2}\left(\kappa \right)\right)$, of the RMSE of *f* and $D_{a}$, respectively. The highest improvement of DDE with respect to SDE is associated with low $c_{2}$ values, i.e. high orientation dispersion. Additionally, for highly aligned voxels the performances of both schemes becomes similar.

## 5 DISCUSSION

Our work shows that modifying the diffusion MRI pulse sequence can mitigate the degeneracy on NODDIDA’s parameter estimation. Our proposal circumvents the need of presetting diffusivities to *a priori* values as in NODDI. We showed that estimating the NODDIDA model through SDE is generally an ill‐posed problem. Depending on the specific combination of model parameters, multiple parameter sets may produce the same signal profile. We show analytically that DDE makes parameter estimation well‐posed, and illustrate for a particular model instance the better behaved cost function obtained with DDE. *In silico* experiments over a wide range of model parameter combinations confirmed that extending the acquisition to DDE makes the inverse problem well‐posed and solves the degeneracy in the parameter estimation. Combining DDE parallel (i.e. LTE) and perpendicular (i.e. PTE) direction pairs not only provides more stable parameter estimates but also increases their accuracy and precision.

In Section 2, we showed that considering a noise‐free scenario, in the case of fibers following a Watson ODF with known (nonzero) concentration parameter *κ* (including the case of parallel fibers), the inverse problem of recovering biophysical parameters from SDE measurements is well‐posed. This is not the case for arbitrary unknown concentration *κ*, where Jelescu et al^17^ first showed experimentally that the parameter estimation from SDE with intermediate b‐values was degenerated. This was analyzed in Jespersen et al^49^ showing that there were two nonlinear equations providing possible solutions. We demonstrated that in the absence of noise the number of BP parameter sets that describe the signal equally well up to $O(b^{2})$ can be either 1, 2, or 4. In contrast, we showed analytically that the **C** tensor includes non‐symmetric independent components that are accessible by DDE, but not by SDE, allowing the complete inverse mapping between the cumulant signal representation and BP parameter space. Consistently, the first experiment showed that in both of the PLIC synthetic voxels, DDE leads to more accurate and precise parameter estimations. This is clearly seen when analyzing the optimization cost‐function which shows that although DDE also presents multiple local minima, the global minimum is substantially deeper, unlike SDE, thus it can be picked in typical noise levels. However, two points in the 5D model parameter space are insufficient to draw more general conclusions. Therefore, the second experiment swept the parameter space extensively using a regular grid. Mean results (see Table 3) showed the minimum RMSE for an acquisition consisting of both linear and planar b‐tensors, suggesting that the optimal combination for the scenario considered is between ${\text{DDE}}_{40\;\;+\;\;20}$ and ${\text{DDE}}_{30\;\;+\;\;30}$ configurations.

Increasing the total number of measurements and SNR will have a larger impact in enhancing DDE parameter estimation than with SDE, since the bimodality present in SDE implies a non‐zero lower bound for the achievable MSE even without noise. Results from^34^ show that the addition of STE data also leads to an increase in the precision of $D_{a}$ and *f* in *in vivo* experiments. In our synthetic experiments the addition of PTE data reduces the RMSE in all the parameter estimates (to a lesser extent in *f* and $D_{e}^{⊥}$). Recently, Dhital et al^35^ showed through *in silico* experiments that incorporating PTE data to LTE data enabled us to discriminate spurious solutions in the cost‐function. This latter result is explained by our theoretical analysis in Section 5 where we derive the independent equations provided by DDE that make the inverse problem well‐posed. While finalizing this paper, a preprint^62^ appeared, reaching similar conclusions.

Biophysical models are promising for extracting microstructure‐specific information but care must be taken when applying them in dMRI. Some assumptions are more meaningful than others and hence their impact on parameter estimation must be assessed.^9^ Invalid assumptions in the model will likely produce bias in the resulting microstructural information, which is epistemic and thus such biases cannot be removed simply by removing the degeneracy. Releasing the diffusivities in the typical two‐compartment model eliminates an invalid assumption, reduces possible biases in the estimated parameters, and provides extra information amenable to be used as a biomarker of microstructural integrity and sensitive to specific disease processes.^44^, ^45^, ^63^ In this work, we have focused on analyzing the estimability of the model under different acquisition settings. The validation against complementary real data is an independent problem. Both should be addressed further to bring biophysical models to the clinic.

Jespersen et al^19^ showed the estimation of the SM was stable and without degeneracy when using extremely high b‐values ($15\;\text{ms}/\mu {\text{m}}^{2}$) on *ex vivo* data. Recent work by Novikov et al^18^ studied the unconstrained SM and concluded that if high b‐values are unfeasible then orthogonal measurements might be an alternative to uniquely relate the kernel parameters to the signal. Veraart et al extended the SM to acquisitions with varying echo time (TE).^64^ This latter work goes in a similar direction to our work here, i.e. adding extra dimensions to the experiment and changing the objective function to avoid ill‐posedness. However, measuring multiple directions while varying the TE implies increasing the acquisition time and TE, affecting the SNR. However, this approach can be combined with DDE leading to a DDE acquisition with multiple TEs. Recently, Lampinen et al^15^ showed that by acquiring data with linear and spherical tensor encodings the accuracy in estimating the microstructural anisotropy was increased compared to that derived from NODDI’s parameters. Additionally, Dhital et al^41^ measured the intracellular diffusivity using isotropic encoding. These two works point in a similar direction than ours, i.e. extending the acquisition to combine different shapes of b‐tensors to maximize accuracy and precision. Future work will study the generalization of the model to a multidimensional diffusion acquisition, since the **C** tensor can be fully sampled using different combinations of b‐tensor shapes, not only by LTE + PTE. Also, a detailed analysis of the impact of noise will be performed, further assessing the practical identifiability of the model parameters.

This work’s aim was to demonstrate that it is possible to solve the intrinsic degeneracy of the SM with a Watson fODF using DDE. Although a cylindrically symmetric fODF might be insufficient to model crossing fibers, it may provide a reasonable approximation in the spinal cord and certain other white matter fiber bundles,^65^ or in highly dispersed tissues like gray matter. Work by Tariq et al has extended the initial NODDI model to a Bingham ODF.^66^ Additionally, Novikov et al^18^ proposed the unconstrained SM with ODF to be described by a series of spherical harmonics. We plan to extend the analysis in this paper to general ODFs. The extension of biophysical models to multidimensional dMRI acquisitions^32^ should be further explored. The comparisons made in this work between SDE and DDE protocols do not consider the optimization of the diffusion directions in DDE, just taking four arbitrary chosen DDE protocols extrapolated from an optimized SDE. We expect that further optimization of the DDE acquisition protocol may also lead to larger improvements. Finally, the largest errors in the parameter estimates occur for *κ* → 0. This might mean that for highly dispersed tissue (i.e. grey matter) many measurements might be needed to accurately estimate model parameters.

## 6 CONCLUSIONS

The potential increase in sensitivity and specificity in detecting brain microstructural changes is a major driving force for developing biophysical models. However, non‐linear parameter estimation of these models is not necessarily well‐posed and can lead to unreliable parameter values. In this work, we not only extended the NODDIDA biophysical model from SDE to DDE schemes, but also demonstrated theoretically the advantages this latter approach has. We illustrated how DDE resolves the degeneracy issue intrinsic to this model estimation from SDE. We prove theoretically that DDE provides complementary information that makes the parameter estimation well‐posed. Additionally, this extension leads to an increase in the accuracy and precision in the model parameter estimates in the presence of noise. The combination of parallel and perpendicular measurements for optimal parameter estimation as function of SNR and measurement time remains to be investigated. Our approach does not require high diffusion weightings to make the inverse problem well‐posed and it can be further developed for the unconstrained SM.

## NOTE

1 These equations are presented in Ref. 49 (Equation 9). However, in their expression, $\Delta_{e}$ incorrectly appears squared also in the second term of the equation for $W\left(\xi \right){\overset{¯}{D}}^{2}$. This was a typing error.

## APPENDIX A

### Inverting the Full‐System for the Watson Case

The system in Equation 8 has a unique solution as long as *κ* ≠ 0 ($\det L\;=\;p_{2}$ and $\det M\;=\;-\frac{1}{2}p_{2}p_{4}$). Their inverse matrices are:

(A1) $$\begin{array}{cc} L^{-1}\;=\;\frac{1}{p_{2}}\left[\begin{array}{cc} 1 & -1\\ -\frac{1}{3}\;+\;\frac{1}{3}p_{2} & \frac{1}{3}\;+\;\frac{2}{3}p_{2} \end{array}\right]\\ M^{-1} & \;=\;\frac{-4}{p_{2}p_{4}}\left[\begin{array}{ccc} -p_{2}/2 & -p_{2}/2 & p_{2}\\ \frac{3}{14}p_{2}-\frac{5}{56}p_{4} & \frac{3}{14}p_{2}\;+\;\frac{9}{56}p_{4} & -\frac{3}{7}p_{2}-\frac{1}{14}p_{4}\\ -\frac{3}{70}p_{2}\;+\;\frac{5}{84}p_{4}-\frac{1}{60}p_{2}p_{4} & -\frac{3}{70}p_{2}-\frac{3}{28}p_{4}-\frac{1}{10}p_{2}p_{4} & \frac{3}{35}p_{2}\;+\;\frac{1}{21}p_{4}-\frac{2}{15}p_{2}p_{4} \end{array}\right]. \end{array}$$

These provide expressions for *α*,* β*,* γ*,* δ* and *ε* that only depend on *κ* and the DK parameters.

## APPENDIX B

### Invariant Decomposition of an Axially Symmetric Fourth Order Tensor

In Hansen et al^51^ the invariant decomposition of a fully symmetric fourth‐order tensor with axial symmetry is presented. Here we follow an analogous procedure for the decomposition of a fourth order tensor, **C**, with only major and minor symmetries.

Any axially symmetric tensor can be expressed in terms of two ingredients: the symmetry axis vector, ***μ***, and the isotropic tensor (Kronecker delta), $\delta_{\mathrm{ij}}$, which can be grouped by the number of copies of ***μ*** included. For a fourth order tensor, this gives **C** = **E** + **F** + **G**, where:

(B1) $$\begin{array}{cc} E_{ijk\ell } & \;=\;a\mu_{i}\mu_{j}\mu_{k}\mu_{\ell },\\ F_{ijk\ell } & \;=\;b\mu_{i}\mu_{j}\delta_{k\ell }\;+\;c\mu_{k}\mu_{\ell }\delta_{\mathrm{ij}}\;+\;d\mu_{i}\mu_{k}\delta_{j\ell }\;+\;e\mu_{j}\mu_{k}\delta_{i\ell }\;+\;f\mu_{i}\mu_{\ell }\delta_{\mathrm{jk}}\;+\;g\mu_{j}\mu_{\ell }\delta_{\mathrm{ik}},\\ G_{ijk\ell } & \;=\;h\delta_{\mathrm{ij}}\delta_{k\ell }\;+\;m\delta_{\mathrm{ik}}\delta_{j\ell }\;+\;n\delta_{i\ell }\delta_{\mathrm{jk}}, \end{array}$$

are linear combinations of one term with four times ***μ***, six terms including it twice, and three terms not including it, respectively. Imposing the major and minor symmetries of the tensor **C**, we find the constraints *a*,* b* = *c*,* d* = *e* = *f* = *g*,* h*, and *m* = *n*, resulting in only five independent components:

(B2) $$\begin{array}{cc} E_{ijk\ell } & \;=\;a\mu_{i}\mu_{j}\mu_{k}\mu_{\ell },\\ F_{ijk\ell } & \;=\;b\left(\mu_{i}\mu_{j}\delta_{k\ell }\;+\;\mu_{k}\mu_{\ell }\delta_{\mathrm{ij}}\right)\;+\;d\left(\mu_{i}\mu_{k}\delta_{j\ell }\;+\;\mu_{j}\mu_{k}\delta_{i\ell }\;+\;\mu_{i}\mu_{\ell }\delta_{\mathrm{jk}}\;+\;\mu_{j}\mu_{\ell }\delta_{\mathrm{ik}}\right),\\ G_{ijk\ell } & \;=\;h\delta_{\mathrm{ij}}\delta_{k\ell }\;+\;m\left(\delta_{\mathrm{ik}}\delta_{j\ell }\;+\;\delta_{i\ell }\delta_{\mathrm{jk}}\right), \end{array}$$

Finally, splitting each of the three tensors above into its symmetric and complementary parts we get:

(B3) $$C\;=\;aP\;+\;\left(2b\;+\;4d\right)Q\;+\;\left(h\;+\;2m\right)I\;+\;\frac{4}{3}\left(b-d\right)R\;+\;\frac{3}{2}\left(h-m\right)J,$$

where

(B4) $$\begin{array}{cc} P_{ijk\ell } & \;=\;\frac{1}{a}E_{\left(ijk\ell \right)},\;\\ Q_{ijk\ell } & \;=\;\frac{1}{2b\;+\;4d}F_{\left(ijk\ell \right)},\;I_{ijk\ell }\;=\;\frac{1}{h\;+\;2m}G_{\left(ijk\ell \right)},\\ R_{ijk\ell } & \;=\;\frac{3}{4(b-d)}\left(F_{ijk\ell }-F_{\left(ijk\ell \right)}\right),\;J_{ijk\ell }\;=\;\frac{3}{2(h-m)}\left(G_{ijk\ell }-G_{\left(ijk\ell \right)}\right), \end{array}$$

result in the tensors defined in Equation 18.

## APPENDIX C

### Single Solution for *κ* from DDE

From the systems in Equations 8 and 24, we can select 4 equations generating the joint system:

(C1) $$\left[\begin{array}{cc} h_{4}\left(1,\kappa \right)2h_{2}\left(1,\kappa \right) & 1\\ h_{4}\left(0,\kappa \right)2h_{2}\left(0,\kappa \right) & 1\\ 0h_{2}\left(1,\kappa \right)-h_{2}\left(0,\kappa \right) & 0\\ 02h_{2}\left(0,\kappa \right) & 1 \end{array}\right]\left[\begin{array}{c} \gamma \\ \delta \\ \epsilon \end{array}\right]\;=\;\left[\begin{array}{c} \frac{1}{3}W_{\|}{\overset{¯}{D}}^{2}\;+\;D_{\|}^{2}\\ \frac{1}{3}W_{⊥}{\overset{¯}{D}}^{2}\;+\;D_{⊥}^{2}\\ \frac{3}{4}\zeta_{1}\;+\;D_{⊥}D_{\|}-D_{⊥}^{2}\\ \frac{3}{2}\zeta_{2}\;+\;D_{⊥}^{2} \end{array}\right]$$

By simple linear combinations we reach  Finally, dividing the first and second equations, the dependency on *γ* cancels out, resulting in Equation 25, providing a single solution for *κ*. This is possible since *γ* is strictly positive, unless there are no sticks (*f* = 0) and the extracellular diffusion is isotropic ($\Delta_{e}\;=\;0$), or if there is no extra‐neurite fraction (*f* = 1) and no axial diffusion inside sticks ($D_{a}\;=\;0$), and $h_{4}\left(0,\kappa \right)\;>\;0$ for all finite *κ*.
